# Improvement of Pulmonary Photodynamic Therapy: Nebulisation of Curcumin-Loaded Tetraether Liposomes

**DOI:** 10.3390/pharmaceutics13081243

**Published:** 2021-08-12

**Authors:** Jennifer Lehmann, Michael R. Agel, Konrad H. Engelhardt, Shashank R. Pinnapireddy, Sabine Agel, Lili Duse, Eduard Preis, Matthias Wojcik, Udo Bakowsky

**Affiliations:** 1 Department of Pharmaceutics and Biopharmaceutics, University of Marburg, Robert-Koch-Str. 4, 35037 Marburg, Germany; jennifer.lehmann@pharmazie.uni-marburg.de (J.L.); michael.agel@pharmazie.uni-marburg.de (M.R.A.); konrad.engelhardt@pharmazie.uni-marburg.de (K.H.E.); shashank.pinnapireddy@pharmazie.uni-marburg.de (S.R.P.); lili.s@hotmail.de (L.D.); eduard.preis@pharmazie.uni-marburg.de (E.P.); matthias_wojcik@web.de (M.W.); 2 Imaging Unit, Biomedical Research Center (BFS), University of Giessen, Schubertstr. 81, 35392 Giessen, Germany; sabine.agel@bfs.uni-giessen.de

**Keywords:** tetraether liposomes, nebulisation, liposomal stability, photodynamic therapy, curcumin, A549 cells

## Abstract

Lung cancer is one of the most common causes for a high number of cancer related mortalities worldwide. Therefore, it is important to improve the therapy by finding new targets and developing convenient therapies. One of these novel non-invasive strategies is the combination of pulmonary delivered tetraether liposomes and photodynamic therapy. In this study, liposomal model formulations containing the photosensitiser curcumin were nebulised via two different technologies, vibrating-mesh nebulisation and air-jet nebulisation, and compared with each other. Particle size and ζ-potential of the liposomes were investigated using dynamic light scattering and laser Doppler anemometry, respectively. Furthermore, atomic force microscopy and transmission electron microscopy were used to determine the morphological characteristics. Using a twin glass impinger, suitable aerodynamic properties were observed, with the fine particle fraction of the aerosols being ≤62.7 ± 1.6%. In vitro irradiation experiments on lung carcinoma cells (A549) revealed an excellent cytotoxic response of the nebulised liposomes in which the stabilisation of the lipid bilayer was the determining factor. Internalisation of nebulised curcumin-loaded liposomes was visualised utilising confocal laser scanning microscopy. Based on these results, the pulmonary application of curcumin-loaded tetraether liposomes can be considered as a promising approach for the photodynamic therapy against lung cancer.

## 1. Introduction

Cancer remains as one of the major challenges in healthcare worldwide and is currently responsible for the majority of global deaths [1]. In recent years, lung cancer was the most common type of cancer and with 18% of the total cancer deaths, it was the leading cause of cancer death in both sexes combined [2]. According to the type of lung cancer and individual risk assessment, standard therapeutic strategies involve chemotherapy, radiotherapy, a combination of both or less frequently, treatment with monoclonal antibodies [3,4]. One of the basic problems in cancer therapy is the high rate of side effects due to undesired tissue destruction and a loss of anatomical and physiological integrity [5]. Therefore, scientists aim to improve the current treatment guidelines as well as to develop treatments with novel mechanisms and targets.

One of these promising mechanisms is the application of light, which itself has a long tradition in the history of medicine, in combination with a photoactive drug collectively termed photodynamic therapy (PDT). The basic principle of PDT is a combination of three otherwise non-toxic components, a photosensitiser, light and oxygen [6,7]. The photosensitiser is a molecule that can be excited by light, mostly due to a conjugated π-electron system. Upon excitation with light of a specific wavelength, the photosensitiser generates reactive oxygen species (ROS) in the presence of oxygen, thereby exhibiting a cytotoxic effect towards surrounding cancer cells [8,9]. The emerged oxygen species has a half-life of only 0.01–0.04 µs and a restricted site of action of 0.01–0.02 µm [8,10]. Due to this, an effect occurs only within a very small range to the irradiation site [11]. Additionally, PDT is able to damage tumour-associated vasculature, resulting in an anaemic infarction of the tumour and can activate the innate immune response against cancer cells. The three mechanisms of PDT can act synergistically and influence each other [12,13]. Other advantages of the PDT are a minor impact towards healthy tissues, its effectiveness against chemo-resistant cancer cell types and being combinable with other therapies such as conventional chemotherapy, microbial adjuvants or cytokines for a T-cell therapy [12,14,15].

There are many different photosensitisers that have been tested in studies, but only very few have reached the stage of advanced human clinical trials or approval for clinical use [14,16]. One such photosensitiser with a huge therapeutic potential is curcumin [17,18,19]. It is a polyphenol extracted from the rhizomes of turmeric (*Curcuma longa*), naturally occurring in south and southeast Asia [20]. Curcumin has an orange-yellow colour due to its phenylogous π-electron system, and is the most active ingredient of turmeric making approximately 0.5–3% of its weight [14]. Since several decades, it has been an object to pharmaceutical research due to its anti-inflammatory, anti-oxidative, anti-microbial and anti-carcinogenic effects [21,22,23]. However, curcumin is prone to an extensive first-pass metabolism after oral administration, undergoes a fast metabolic reduction and is subject to biliary excretion [24]. Besides, due to the highly lipophilic properties of this photosensitiser, with a solubility of merely 11 ng/mL in phosphate buffer saline (pH 5), it has a very limited bioavailability [24,25].

These limitations can be overcome by the liposomal encapsulation of curcumin. Within the lipid bilayer of the liposomes, the active ingredient is protected from the physiological degradation on the one hand [9], and the cellular uptake as well as bio-membrane permeability are enhanced on the other hand [9,26]. Altogether, bioavailability, biocompatibility and the circulation time can be improved through liposomal encapsulation, enhancing the overall effectiveness of the photosensitiser [27].

Nebulisers have turned into a trendsetting option in the treatment of a multitude of diseases due to increased patient compliance. For instance with the usage of glycerosomes and hyalurosomes, which are stable towards the process of nebulisation and proved to be good vehicles for a pulmonary application [28,29]. The nebulisation of liposomes can also lead to a considerable improvement in lung cancer disease management.

The major focus of this study was to investigate a non-invasive convenient approach for the treatment of lung cancer by combining nebulised liposomes and PDT. For this purpose, behaviour and stability of three liposomal model formulations were investigated before and after the process of nebulisation and their in vitro efficacy was assessed using A549 lung adenocarcinoma cells. The liposomal formulations had different degrees of membrane stabilisation, amongst others through the usage of bipolar tetraether lipids extracted from the biomass of the archaea *Sulfolobus acidocaldarius*. This plays an important role in membrane integrity and thus the ability to successfully deliver the active ingredient. Curcumin was chosen as it is a commonly used photosensitizer with a well-known impact [14,17,30]. Additionally, the suitability of two different nebulisation techniques, vibrating-mesh and air-jet, was tested and compared for the nebulisation of liposomes in small amounts.

## 2. Materials and Methods

### 2.1. Materials

Curcumin (≥80%), cholesterol (≥99%), HEPES (≥99.5%), ethanol (absolute, ≥99.8%), D-(+)-trehalose dehydrate (≥99%) and 3-(4,5-dimethylthiazol-2-yl)-2,5-diphenyltetrazolium bromide (MTT) were obtained from Sigma-Aldrich Chemie GmbH (Taufkirchen, Germany). The lipids 1,2-dipalmitoyl-sn-glycero-3-phosphocholine (DPPC) and 1,2-dioleoyl-sn-glycero-3-phosphoethanolamine (DOPE) were a gift from Lipoid GmbH (Ludwigshafen am Rhein, Germany). Fractions containing the polar tetraether lipids caldarchaeol (GDGT, approx. 10% of the extract) and calditoglycerocaldarchaeol (GDNT, approximately 90% of the extract) were extracted from the biomass of *Sulfolobus acidocaldarius* (Transmit GmbH, Gießen, Germany) [31]. The ends of GDNT contain phosphatidylmyoinositol and β-glucose respectively while GDGT contains phosphatidylmyoinositol and β-D-galactosyl-D-glucose on either ends respectively [32]. Ultrapure water from PURELAB^®^ flex 4 (ELGA LabWater, High Wycombe, UK) was used for all experiments in this study. All solvents used were of analytical or HPLC grade.

### 2.2. Cell Culture

A549 adenocarcinomic human alveolar basal epithelial cells were obtained from ATCC (American Type Culture Collection, Manassas, VA, USA). The cells were cultured in DMEM (Capricorn Scientific GmbH, Ebsdorfergrund, Germany) supplemented with 10% foetal bovine serum (Capricorn Scientific GmbH). The cells were cultivated at 5% CO_2_ and 37 °C under humid conditions. They were grown as monolayers and passaged upon reaching 80% confluency.

### 2.3. Irradiation Device

For photodynamic activation, a low power prototype LED device consisting of an array of light emitting diodes designed to fit multiwall plates was used. The device was custom manufactured by Lumundus GmbH (Eisenach, Germany), equipped with two different arrays of LEDs capable of emitting light at different wavelengths. Activation took place at 457 nm, the actual fluence used in this study was calculated based on the amperage and irradiation time [26].

### 2.4. Preparation of Liposomes

Liposomes were prepared via thin-film-hydration method using stock solutions of curcumin in ethanol and lipids dissolved in chloroform:methanol (2:1 (*v*/*v*)). The ingredients were mixed in a 10 mL round bottom flask with a curcumin to lipid ratio of 1:10. To gain a thin film, the mixture was evaporated at 40 °C using a Laborota 4000 rotary evaporator (Heidolph Instruments, Schwabach, Germany) fitted with a vacuum pump (KNF Neuberger GmbH, Freiburg im Breisgau, Germany). Afterwards, the film was rehydrated with HEPES (20 mM, pH 7.4) to gain a final concentration of 1 mg/mL. The liposomal formulations were sonicated in a bath sonicator (Elamsonic P30H, Elma Schmidbauer GmbH, Singen, Germany) above the phase transition temperature (T_c_) for 30 min.

To homogenise the vesicles, all liposomes were extruded with an Avanti Mini Extruder (Avanti Polar Lipids, INC., Alabaster, AL, USA) using polycarbonate membranes with a pore size of 200 nm. This step was carried out above the T_c_. The obtained liposomes were stored at 4 °C.

In total three formulations were compared with each other in this study (Table 1).

### 2.5. Lyophilisation

To improve long-term storage stability, the liposomes were lyophilised using an Alpha 1-4 LSC freeze-dryer (Martin Christ, Osterrode am Harz, Germany). In this process, D-(+)-trehalose dihydrate was used to protect the liposomes from collapsing or aggregating during lyophilisation.

Briefly, the liposomal formulations were prepared as stated in Section 2.4 and rehydrated in filtrated HEPES containing 4 mg/mL D-(+)-trehalose dihydrate. A total of 500 µL of the freshly produced liposomes in round bottom flasks were rapidly frozen by shaking in liquid nitrogen to increase the surface and lyophilised with the following parameters: primary drying for 24 h at +15 °C shelf temperature and a partial vacuum of 0.120 mbar, followed by secondary drying for 24 h at +25 °C shelf temperature and 0.100 mbar. The lyophilised residual underwent visual examination. Prior to use and nebulisation with PARI VELOX^®^, the freeze-dried liposomes were redispersed in ultrapure water.

### 2.6. Nebulisation, Aerosol Output and Emitted Volume

For the nebulisation of previously produced liposomes, two nebulisers representing two different techniques of nebulisation were used and compared: PARI VELOX^®^ (PARI GmbH, Starnberg, Germany), a metal-based vibrating-mesh nebuliser with a resonance frequency of 160 kHz and PARI BOY^®^ SX utilising compressed air with an operating pressure of approximately 1.6 bar and a nozzle size of 0.48 mm. Briefly, 2 mL of each liposomal formulation were pipetted in the sample reservoir and collected again after nebulisation directly into 5 mL tubes (Sarstedt AG & Co. KG, Nümbrecht, Germany). The nebulisation time and volume of nebulised liposomes were measured for each formulation to calculate the aerosol output rate (L/min) and emitted volume (%). All formulations were nebulised at a total lipid concentration of 1 mg/mL.

### 2.7. Dynamic Light Scattering and Laser Doppler Anemometry

Particle size (hydrodynamic diameter applying intensity mode) and PdI (polydispersity index) of all liposomal formulations were measured by dynamic light scattering (DLS) using a Zetasizer Nano ZS (Malvern Instruments, Kassel, Germany). All measurements were carried at a wavelength of 633 nm (HeNe laser) and 20.5 °C. For this purpose, a clear disposable folded capillary cell (DTS1060, Malvern Instruments, Kassel, Germany) was utilised. The instrument adjusted attenuation and measurement position of the laser automatically; the detection angle was 173°. Immediately before measurement, 900 µL liposomes were diluted with 100 µL HEPES (20 mM, pH 7.4), thus at the ratio of 1:10. All samples were measured three times with the instrument performing 3 runs with 15 sub-runs each, all sub-runs lasting 10 s.

Laser Doppler anemometry (LDA) was used to determine the ζ-potential with the Zetasizer Nano ZS. The liposomes were diluted as described above. All samples were measured three times and the instrument performed automatically 15–100 sub-runs. Values from three independent samples were considered.

### 2.8. Encapsulation Efficiency and Loading Capacity

To extract curcumin from the liposomes, the method of Duse et al. was applied [14,26]. Briefly, 300 µL of each formulation were centrifuged for 90 min at 2000× *g* (Eppendorf 5418 Centrifuge, Eppendorf AG, Hamburg, Germany) following further centrifugation steps to remove all non-dissolved lipids. The pellet was dissolved in 200 µL ethanol and 200 µL HEPES (20 mM, pH 7.4), 200 µL of the supernatant was mixed with 200 µL ethanol. Subsequently, curcumin in all samples was quantified spectrophotometrically at a wavelength of 425 nm using Multiskan GO plate reader (Thermo Fischer Scientific GmbH, Dreieich, Germany). The required calibration curve was generated with predetermined concentrations of curcumin in the same solvent composition and unloaded liposomes were used as blank. Calculations of the encapsulation efficiency (EE) and loading capacity (LC) were then carried out with the following equations:

(1) $$\mathrm{EE}\left[\%\right]=\frac{{\mathrm{Curcumin}}_{\;\mathrm{encapsulated}}}{{\mathrm{Curcumin}\;}_{\mathrm{total}}}\times 100$$

(2) $$\mathrm{LC}\left[\%\right]=\frac{{\mathrm{Curcumin}\;}_{\mathrm{encapsulated}}}{{\mathrm{Ingredients}}_{\;\mathrm{total}}}\times 100$$

### 2.9. Atomic Force Microscopy

The morphological characteristics of the liposomal formulations were studied with atomic force microscopy (AFM) using a NanoWizard^®^ 3 (JPK Instruments AG, Berlin, Germany) with silicon cantilevers (HQ:NSC14/AL BS, Mikromasch Europe, Wetzlar, Germany). The measurements were carried out using intermittent contact mode at scan rates between 0.5 to 1 Hz to avoid damaging the lipid membrane [33]. A resonance frequency of 120 kHz and the force constant 5 N/m was used. Different formulations of nebulised liposomes were diluted 1:10 with ultrapure water, 10 µL of the mixture was placed onto glass slides and left to dry at room temperature. To gain the final images, the amplitude signal of the cantilever in the trace direction and the height signal in retrace direction were used and the images were processed using JPKSPM data processing software (JPK Instruments AG).

In addition, AFM images were analysed for size confirmation. For this purpose, the average liposomal size was determined by analysing a representative number of height images using ImageJ software (v1.52a, National Institutes of Health, Bethesda, MD, USA) [33,34].

### 2.10. Transmission Electron Microscopy

As a second method to examine the liposomal morphology, transmission electron microscopy (TEM) was used. The sample preparation was carried out on 400 mesh copper grids, coated with 1.2% Formvar and carbon (Plano GmbH, Wetzlar, Germany). Briefly, the liposomes were diluted to 1:10 ratio with HEPES (20 mM, pH 7.4) and 10 µL were placed on a grid. After 5 min of incubation, the liquid was withdrawn by suction with a Whatman 4 filter paper (Whatman plc, Maidstone, UK). Then the grids were placed for 5 min on 20 µL 2% uranyl acetate, which was used as contrast agent for the negative staining. The liquid was withdrawn by suction again and the grids were placed on 20 µL H_2_O as a washing step. After the remaining liquid was carefully removed, the grids were left to dry. To obtain the images, samples were examined using a Leo 912 AB TEM (Carl Zeiss Microscopy GmbH, Jena, Germany) with different magnifications and an accelerating voltage of 100 kV.

### 2.11. Aerodynamic Properties

To define the aerodynamic properties, a determination of the fine particle fraction (FPF) was carried out according to monograph 2.9.18 with device A (twin glass impinger) mentioned in the European Pharmacopoeia 9.5. Briefly, 7 mL and 30 mL of water:acetonitrile (1:1 (*v*/*v*)) were added to the top and bottom chamber, respectively. The device was assembled and connected to an Erweka vacuum pump VP 1000 (Erweka GmbH, Heusenstamm, Germany). Using a flow meter DFM 2000 (Copley Scientific, Nottingham, UK), the flow rate was adjusted to 60 ± 5 L/min. The liposomal formulations were pipetted into the sample reservoir of a PARI VELOX^®^ nebuliser, which was linked to the glass twin impinger (Hannes & König GmbH, Heusenstamm, Germany) via mouthpiece. The vacuum pump was turned on 10 s before starting the nebulisation. After 60 s, the nebulisation process was stopped, and the vacuum pump was turned off 5 s afterwards. The amount of curcumin in each chamber was quantified spectrophotometrically. FPF was then calculated according to the following equation:

(3) $$\mathrm{FPF}\left[\%\right]=\frac{{\mathrm{Curcumin}\;}_{\mathrm{bottom}\;\mathrm{chamber}}}{{\mathrm{Curcumin}}_{\;\mathrm{total}\;}}\times 100$$

### 2.12. Mucous Membrane Compatibility

The determination of mucosal tolerance for all liposomal formulations was performed by HET-CAM assay [35]. The chorio-allantoic membrane (CAM) is an extraembryonic membrane that results from the fusion of the shell membrane (chorion) with the allantoic cavity (rectal protrusion of the embryo) in a fertilized chicken egg [36]. For preparation, the fertilized chicken eggs had to be thoroughly cleaned with ethanol 70% (*v*/*v*) after delivery and incubated at 37.8 °C and 60% relative humidity in a Thermo de Luxe 250 incubator (Hemel Brutgeräte GmbH). Eggs were continuously turned 12 times per day until egg development day (EDD) 3. On EDD 3, a round incision (⌀ 28 mm) was added to the blunt side of the eggs with the aid of an EggPunch pneumatic egg opener (Schuett-Biotec GmbH). An opening was then created by carefully removing the shell as well as the shell membrane with curved Dumostar Style 7 forceps (Manufactures D’Outils Dumont SA). For protection, the opened eggs subsequently had to be covered with a sterile Petri dish and incubated in a vertical position in the incubator.

On EDD 9300 μL of nebulised liposomes were applied to the CAM and observed during 5 min under a Stemi 2000-C stereomicroscope (Carl Zeiss AG) at 13-fold magnification. The study was performed with 6 eggs per sample. HEPES (20 mM, pH 7.4) served as a negative control, sodium dodecyl sulfate (SDS) 1% (m/m) and 0.1 N sodium hydroxide were used as positive control. The determination was based on stereomicroscopic images taken with a Moticam 5 MP digital camera (Motic Deutschland GmbH) and an irritation score (*IS*), which included an assessment of haemolysis (*H*), vessel lysis (*L*), and blood coagulation (*C*) [37]. Events were photographed at the time of their occurrence and the irritation score was calculated as follows according to the literature [38]:

(4) $$IS=\frac{\left(301-H\left[s\right]\right)}{300}\times 5+\frac{\left(301-L\left[s\right]\right)}{300}\times 7+\frac{\left(301-C\left[s\right]\right)}{300}\times 9\;$$

### 2.13. In Vitro Cytotoxicity

A549 cells were seeded onto 96-well plates (NUNC, Thermo Fischer Scientific GmbH, Dreieich, Germany) with a seeding density of 1 × 10^4^ cells/0.35 cm^2^ (per well) and the plates were incubated for 24 h. For the in vitro cytotoxicity assay, the cells were treated with various concentrations of all liposomal formulations using a serial dilution starting with 100 µM of curcumin. After 4 h of incubation, liposomal formulations were aspirated and replaced with fresh medium. The cells were irradiated at 457 nm using an LED device (Lumundus GmbH) with a fluence of 6.61 J cm^−2^. After 24 h, the cells were incubated with MTT reagent (0.2 mg/mL) for 4 h and DMSO was then used to dissolve the resulting formazan crystals. The absorbance was determined at a wavelength of 570 nm using FLUOstar^®^ Optima (BMG Labtech, Ortenberg, Germany). Untreated cells (blank) and an unirradiated microtiter plate (dark plate) were used as controls. The viability of blank cells was considered as 100%.

### 2.14. Cellular Uptake Studies

Liposomal formulations can be absorbed into the cells in different ways and thus develop their effect. For a better understanding of these effects, the uptake pathways were determined. This was done by inhibiting two major pathways respectively, determining the cell survival rate and comparing it to that of untreated cells. Chlorpromazine served as an inhibitor of clathrin-mediated endocytosis, and filipin III inhibited caveolae-mediated endocytosis. As previously described, A549 cells were seeded at a density of 1 × 10^4^ cells per well in 96-well plates and incubated for 24 h. Subsequently, these had to be washed twice with PBS buffer containing Ca^2+^/Mg^2+^ to ensure removal of serum residues that could interfere with the effect of the inhibitors. Then, preincubation of chlorpromazine and filipin III (5 μg/mL each) for 30 min took place, followed by incubation with liposomes (100 μM each) for 4 h. Afterwards, the liposomes were replaced with fresh medium and irradiation of the cells took place at λ = 457 nm and with a fluence of 6.61 J/cm^2^. After further incubation of the cells for 24 h, a determination of cell viability was performed by in vitro cytotoxicity assay as described in Section 2.13. Untreated and treated but unirradiated cells as well as cells treated with inhibitor only were used as controls.

### 2.15. Confocal Laser Scanning Microscopy (CLSM)

A549 cells were seeded onto 15 mm coverslips inside 12-well plates (NUNC, Thermo Fischer Scientific GmbH, Dreieich, Germany) with a seeding density of 9 × 10^4^ cells/3.5 cm^2^ (per well). The cells were treated with liposomal formulations containing 100 µM of curcumin and incubated for 4 h. After irradiation (6.61 J cm^−2^ at 457 nm), the cells were washed twice with PBS buffer containing Ca^2+^ and Mg^2+^ (pH 7.4) and fixed with 4% formaldehyde solution. Afterwards, the cells were washed twice with PBS buffer and the cell nuclei were counterstained with 0.1 µg/mL 4′,6-diamidino-2-phenylindole (DAPI). The cells were then washed with PBS buffer and the coverslips were mounted onto slides and sealed using FluorSave™ (Calbiochem Corp., La Jolla, CA, USA). The cells were examined using an LSM700 confocal laser-scanning microscope (Carl Zeiss Microscopy GmbH, Jena, Germany). All micrographs were recorded at a similar adjustment.

### 2.16. Cellular Migration

The so called “in vitro scratch assay” was performed to determine the growth inhibitory effect of PDT with nebulised liposomes on A549 cells. For this assay, 9 × 10^4^ cells per well were also seeded in 12-well plates and cell growth in the wells was observed until a confluent monolayer was formed (approximately after 48 h). After 4 h of incubation with nebulised liposomes of 100 μM concentration and a subsequent exchange with fresh DMEM medium, it was possible to scratch through the wells with a pipette tip and thus create a gap in the cell monolayer.

### 2.17. Statistical Analysis

All experiments were carried out in triplicates and the values are presented as mean ± standard deviation unless otherwise stated. The statistical significance of measured values was determined by using a two-tailed *t*-test and the probability values of *p* < 0.05 were considered statistically significant.

## 3. Results and Discussion

### 3.1. Suitability of Nebulisers

**Figure 1:**
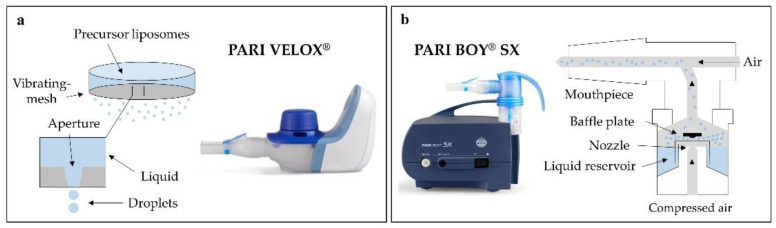
Operating mode of both techniques: (**a**) vibrating-mesh nebulization, (**b**) air-jet nebulisation.

In this study, two nebulisers of different technology were compared to identify the device best suited for the nebulisation of liposomes. PARI VELOX^®^ represents vibrating-mesh nebulisation and PARI BOY^®^ SX air-jet nebulisation. Vibrating-mesh nebulisers extrude liquid through a perforated mesh to generate aerosols, whereas air-jet nebulisers use compressed air to press the liquid through a nozzle of defined size (Figure 1a,b) [39,40].

Generally, PARI VELOX^®^ showed a slightly slower output rate compared to PARI BOY^®^ SX, but a much higher emitted dose. On the vibrating-mesh nebuliser, ultrapure water had a low output rate of 0.27 ± 0.04 mL/min due to a lower concentration of electrolytes [41]. However, the liposomal formulations rehydrated in 20 mM HEPES (pH 7.4) showed output rates of 0.48 ± 0.07 mL/min (DT), 0.50 ± 0.01 mL/min (DC) and 0.53 ± 0.03 mL/min (DD). According to the literature, the average output rate of PARI VELOX^®^ for normal saline (0.9% NaCl) is 0.76 ± 0.18 mL/min [42]. Air-jet nebulisation showed an output rate of 0.29 ± 0.03 mL/min for ultrapure water, 0.65 ± 0.05 mL/min for DT, 0.63 ± 0.03 mL/min for DC and 0.69 ± 0.01 mL/min for DD. The emitted dose of vibrating-mesh nebulisation was 93.5 ± 3.3% (*v*/*v*) for ultrapure water, 94.0 ± 1.5% (*v*/*v*) for DT, 95.5 ± 1.3% (*v*/*v*) for DC and 94.7 ± 1.0% (*v*/*v*) for DD. Air-jet nebulisation revealed an emitted dose of 18.5 ± 2.3% (*v*/*v*) for ultrapure water and 15.3 ± 0.8% (*v*/*v*) (DT), 17.3 ± 1.3% (*v*/*v*) (DC), 14.8 ± 1.5% (*v*/*v*) (DD) for the liposomal formulations. Looking at the liposomal formulations, a slight difference is visible. For both nebulisers, DC had the highest emitted dose. However, the results of all formulations regarding output rate and emitted dose were in the same range for both nebulisers, which was probably because all liposomes were rehydrated with HEPES (20 mM, pH 7.4). The output rate of ultrapure water was much lower and, according to literature, the one of 0.9% NaCl much higher. Thus, an increased conductivity leads to a higher output rate. This confirms previous findings, that the concentration of electrolytes is a significant parameter influencing output rate due to a more efficient liquid breakdown during atomisation [43]. More fluidic liposomes also seem to promote a higher output rate, which can be seen in the case of DD with both nebulisers. It could be explained by smaller and easier separable liposomes which may decrease the liquid resistance.

In the recent literature the two nebulisation techniques are described to function with nanoscaled drug carrier systems [44,45] and are suitable for therapies with small volumes since both require a minimum sample volume of only 2 mL. They provide comparable lung deposition profiles and show reportedly no damage of precursor liposomes [39,45]. A comparison of the measured size values for both nebulisation techniques of all formulations (Table 2) revealed no significant difference (*p* > 0.05) between PARI VELOX^®^ and PARI BOY^®^ SX. PdI and ζ-potential likewise showed no major differences for both nebulisation techniques among all formulations, leading to the assumption that both nebulisers are capable of emitting intact liposomes, suitable for deep lung deposition. However, vibrating-mesh nebulisation seemed more suitable than air-jet nebulisation due to a very high emitted dose and a much better handling with similar output rates. Additionally, PARI VELOX^®^ combines other advantages such as being portable, quiet and cost-effective, making the therapy patient-friendly as well. It was therefore used for all further experiments in this study.

### 3.2. Physicochemical Properties of the Liposomal Formulations

One focus in the course of drug nebulisation was the application of liposomes [39,46]. State of the art in pulmonary drug delivery for the treatment of lung cancer is the nebulisation of different nanocarriers such as nanoparticles, nanoemulsions and liposomes [47,48,49,50]. To achieve a more localised drug delivery, different modifications and targets were tested so far [47,51]. The liposomes tested for the treatment of lung cancer in this study have a great advantage, as the used lipids are biocompatible, biodegradable and present in clinical trials or even approved for clinical use [52]. In the field of pulmonary delivery, several liposomal formulations have been tested in animal studies and clinical trials as well [53].

**Figure 2:**
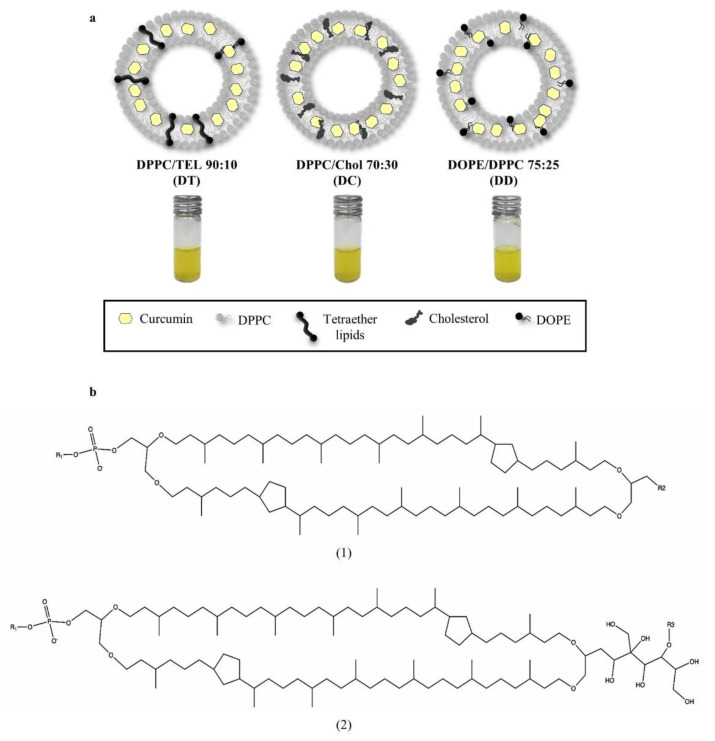
(**a**) Model compositions of liposomes: DT with tetraether lipids providing the highest degree of membrane stabilisation, DC as medium stabilised standard composition with cholesterol and the fluidic composition of DD with no stabilisation of the membrane. (**b**) Structures of the tetraether lipids used in this study. (**1**) GDGT, glycerol dialkylglycerol tetraether and (**2**) GDNT, glycerol dialkylnonitol tetraether with R1: inositol, R2: β-D-glucopyranose and R3: β-D-galactosyl-β-D-glucopyranose.

As there are many suitable lipids for the preparation of liposomes, the present study aimed to compare different representative liposomal compositions. DT (DPPC:TEL 90:10) is the composition with the highest degree of membrane stabilisation as the tetraether lipids are crossing the entire lipid bilayer. This is due to the ring structure of these lipids, originated from four ether bonds (Figure 2b), implementing a tight packing of the lipid bilayer and a rigid behaviour of these liposomes. In addition, TEL reduces liposomal sensitivity towards oxidation [26]. DC (DPPC:Chol 70:30) is a standard medium rigid composition using cholesterol for membrane stabilisation, which is incorporated in the lipid bilayer. The composition DD (DPPC:DOPE 75:25) is more fluidic than the others, with no stabilisation of the membrane (Figure 2a). Upon rehydration of the lipid films, curcumin is packed inside the liposomal bilayer of all formulations having hydrogen bonds to the lipid acyl chains. This encapsulation protects curcumin from degradation and greatly increases its solubility in aqueous media [26,27].

Particle size, PdI and ζ-potential of unnebulised and nebulised liposomal formulations were compared (Table 2), as stable delivery systems are key requirements for a successful application. Looking closer at the results, a few differences between the formulations were noticed. Prior to nebulisation, DT liposomes were slightly larger in diameter than the other formulations due to TEL crossing the lipid bilayer. Particle size and PdI were in the same range before and after nebulisation for this formulation, making it inured to nebulisation. This may be explained by increased packing and stronger hydrogen bonds within the lipid bilayer, reducing the effects of nebulisation on these liposomes [54]. DT also had a more negative ζ-potential compared to the other formulations, which can be explained by the ether bonds of TEL. DC liposomes were smaller than DT, probably due to the smaller cholesterol molecule. Likewise, particle size and PdI were in the same range after nebulisation, revealing them stable to nebulisation as well. Cholesterol can decrease the bilayer fluidity by reducing the movement of the lipid hydrocarbon chains and increase the packing of lipid head groups, which can lead to a smaller particle size [40]. In contrast, DD was a more fluidic formulation due to DOPE, which can destabilise the membrane as it may stimulate a transition from a lamellar to a hexagonal phase [55,56]. This fluidic behaviour could cause the increase in size and PdI after nebulisation, which can be seen in Table 2. Therefore, DD seemed less stable to forces during the process of nebulisation. Liposomal compositions and the total lipid concentration can influence the nebulisation, but conversely, the different nebulisation techniques can have an impact on size, uniformity and integrity of liposomes [57]. It can be seen in this study, that nebulisation with compressed air leads to a lower uniformity of all liposomal formulations. This could be explained by higher shearing forces while pushing the liquid through the nozzle. Both techniques also have a slight impact on liposome sizes and ζ-potentials. The negative ζ-potential of all formulations could be related to the entrapment of curcumin, the different extent seems depending on the liposomal formulation [26].

To increase the storage stability, the three liposomal formulations were lyophilised directly after preparation, rehydrated with ultrapure water and nebulised using PARI VELOX^®^. It was observed that the ζ-potential of these formulations was more negative, which could be explained by a leakage and attachment of curcumin to the outer surface of the liposomes. This observation is confirmed by results of the encapsulation efficiency (Table 3). A change in the ζ-potential can affect the mucoadhesive properties of the liposomes as they are based on the interaction between charged molecules [58]. Size and PdI of all formulations were also increased. Source of this increase could be the cryoprotectant D-(+)-trehalose dehydrate itself, due to its penetration into the liposomes as it leads to a stabilisation of liposomes from inside and outside [59]. According to literature, lyophilising without cryoprotectant leads usually to intense aggregation or fusion [59,60]. Possible causes for the good protective properties of D-(+)-trehalose dehydrate can be the high glass transition temperature and the low tendency to crystallisation [59]. However, lyophilisation prior to nebulisation seemed to have a negative influence on all three formulations.

Altogether, the membrane stabilised formulations DT and DC appeared stable to nebulisation whereas DD as fluidic formulation did not seem eligible.

Generally, liposomes are very convenient as vehicles for all kinds of drugs due to their relatively high encapsulation efficiency [9,26,46]. The lipophilic drug curcumin was used in this study with 100 µg curcumin per 1 mL liposomes. Typically, curcumin is non-uniformly distributed in the lipid bilayer and entirely located inside the hydrophobic interior, which is important for a high drug loading capacity [27]. Previous research indicated that EE and LC are dependent on the composition of lipid acyl chains forming the liposomal bilayer. Lipids containing shorter acyl chains and hence, a lower Tc, are mostly able to incorporate a larger amount of hydrophobic substances such as curcumin due to altered van der Waals linkages [60]. Additionally, unilamellar vesicles, as they emerge after extrusion through polycarbonate membranes, are considered to have better drug incorporation into the bilayer than multilamellar vesicles [46]. These findings stand in accordance with the results of this study, as Table 3 showed relatively high encapsulation efficiencies for all un-nebulised liposomes. Looking closer at the three formulations, a difference in behaviour was visible. Upon addition of TEL, a high encapsulation efficiency to the extent of 93.9% was achieved. This could be due to the fact that TEL, by crossing the lipid bilayer with its acyl chains containing cyclopentane structures, provided a tight membrane packing through more van der Waals linkages. Resultant in a very good capability of encapsulating hydrophobic substances [61,62]. Both, EE and LC seemed quite stable after the process of nebulisation, which speaks again for the high degree of membrane stability as it makes the liposomes less permeable by preserving membrane integrity. Stabilisation through the bipolar structure of TEL also provides thermal stability [63] and better long-term storage stability rates [64]. Besides, TEL can positively affect the intermembrane exchange of the active ingredient and thereby enhance the delivery [64]. The linkages of Cholesterol inside the lipid bilayer of DC similarly promote the encapsulation of curcumin [60], but with 88.4% (Table 3) to a lower extent. DD, which contains the fluidic lipid DOPE, showed the lowest encapsulation efficiency of 85.1%. Compared to the un-nebulised liposomes, EE values of nebulised curcumin-loaded liposomes were decreased. The slight effect of DT can be explained by leftover curcumin attached to the outer surface of the liposomes, remaining even after extrusion [46]. The residual amount of encapsulated curcumin in DC was also relatively high, as the moderate stabilisation with Cholesterol also leads to decreased leakage rates, whereas for DD a much stronger effect was visible probably due to a disintegration of the fluidic liposomes followed by a loss of curcumin. These findings stand in accordance with the ones of DLS analysis as the changes in size, PdI and ζ-potential were correspondent to the different behaviour of the formulations. This is also the case for lyophilised and nebulised curcumin-liposomes, which had the lowest EE and LC. Again, the cryoprotectant D-(+)-trehalose dehydrate could be the cause, as it can replace curcumin inside the liposomal bilayer [58].

With respect to EE and LC of liposomes, another aspect that must be taken under consideration is the state of entrapped curcumin. Hydrophilic drugs encapsulated in the aqueous core have a high tendency to aggregate which can compromise their therapeutic impact, whereas lipophilic drugs such as curcumin have a much lower tendency to aggregate within the bilayer, probably also due to the presence of van der Waals linkages with the lipid acyl chains building a barrier [60].

Again, the membrane stabilisation of DT and DC seemed to be an advantage. The EE and LC of both formulations were high, and the values of DT were quite stable after nebulisation. However, DD, with no membrane stabilisation, exhibited an unstable behaviour since the EE decreased constantly during lyophilisation and nebulisation. The lack of membrane stabilisation revealed a higher loss of entrapped curcumin making DD not suitable for nebulisation after all.

### 3.3. AFM Visualisation

**Figure 3:**
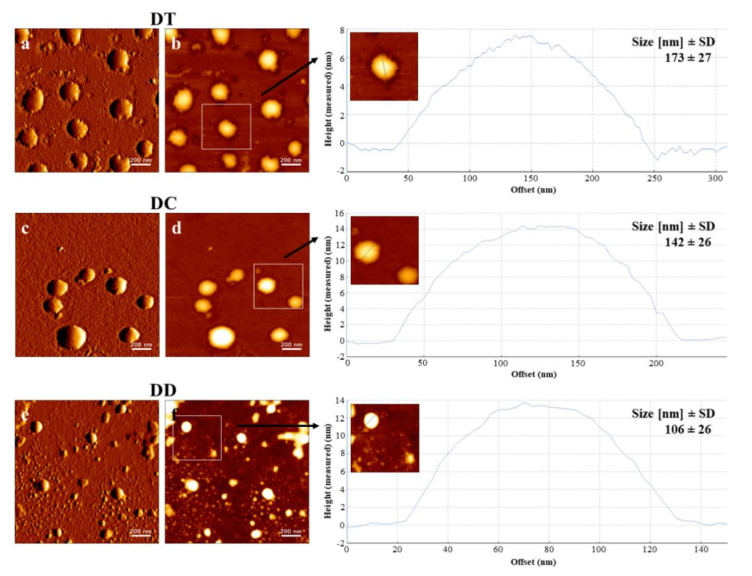
Atomic force microscopy (AFM) visualisation of DT, DC and DD after nebulisation with PARI VELOX^®^, containing a curcumin concentration of 100 µM. Images (**a**,**c**,**e**) are amplitude signals and (**b**,**d**,**f**) are height signals. Liposomes in height images together with their cross-sectional profiles were used for a size evaluation along the shown lines and a morphological characterisation. The stated sizes are mean values ± standard deviation gathered by analysing representative height images using ImageJ software. Scale bars represent 200 nm.

AFM images revealed spherically shaped vesicles for all formulations after nebulisation (Figure 3). Smooth surfaces of the vesicles indicated complete incorporation of curcumin. Images of DD (Figure 3e,f), the most fluidic formulation, exhibited several small spherically shaped vesicles, presumably due to a disintegration through the vibrating mesh during the process of nebulisation. This disintegration could be explained by the fluidic behaviour and positive charge, that both lipids have in this formulation. Conductivity greatly affects the process of nebulisation [65], in this case the charge probably led to an interaction with the surface of the metal-based mesh promoting further disintegration. Another explanation can be the high Tc, originated by long hydrocarbon chains. Below this temperature, liposomes act more like solids than as fluids, increasing the probability of decomposition [60]. This stands in accordance with the results of EE and LC, as DD revealed to be not suitable for nebulisation. In contrast, the highly stabilised formulation DT (Figure 3a,b) and the medium stabilised formulation DC (Figure 3c,d) showed constant behaviour in terms of their size. Although the effectiveness of inhalable therapies depends on the aerodynamic properties of the nebulised vehicles, the output of intact liposomes from the nebuliser, which was achieved in this study, is a fundamental requirement for the applicability of the therapy [66]. Liposomes of all formulations appeared slightly bigger in the AFM images. On the one hand, probably due to spreading on the glass slides while they were left to dry as a part of the sample preparation, on the other hand, due to different measuring conditions compared to DLS. DLS results were obtained in aqueous conditions whereas AFM measurements took place under dry conditions. However, size analysis using the height images (Figure 3) stood in agreement with the results obtained with DLS.

### 3.4. TEM Visualisation

**Figure 4:**
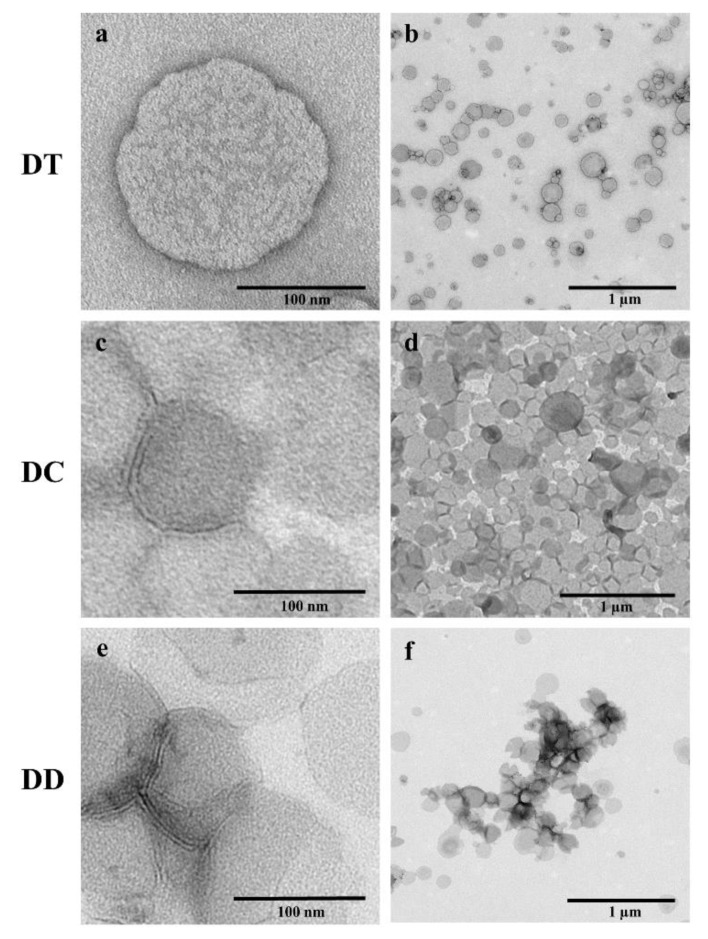
Transmission electron microscope (TEM) images of the liposomal formulations DT, DC and DD after nebulisation, containing a curcumin concentration of 100 µM. The samples were negatively stained with 2% uranyl acetate. Scale bars represent 100 nm for the images (**a**,**c**,**e**) and 1 µm for the images (**b**,**d**,**f**).

Transmission electron microscopy enabled a precise examination of morphological differences between the liposomal formulations after nebulisation with PARI VELOX^®^. Through the method of negative staining with uranyl acetate it was possible to image the liposomes in their original environment, which was HEPES (20 mM, pH 7.4) in this study. Likewise seen in the particular AFM images, spherically shaped vesicles were visible for the three formulations. The most rigid formulation DT appeared as well-defined vesicles, which were slightly dented (Figure 4a,b). This was in accordance with AFM measurements. The liposomal diameter determined from TEM images corresponded to the ones obtained via DLS and AFM. These findings indicated the stability of tetraether liposomes during the process of nebulisation. The appearance of the membrane stabilised DC also matched the one in AFM images, as the vesicles were equal round and with nearly uniform sizes (Figure 4d). Additionally, TEM visualisation revealed the lipid bilayer of DC liposomes in the magnified image (Figure 4c) and hence their intactness. Likewise, the vesicle diameter determined from TEM images corresponded to previous ones indicating DC’s stability towards nebulisation. DD, as the most fluidic formulation in this study, showed different behaviour. A few vesicles were identified as intact liposomes due to the visible lipid bilayer (Figure 4e), but the majority was found to be spread on the grid (Figure 4f). For this reason, the diameter determined from TEM images appeared larger compared to the ones obtained by DLS, excluded the intact vesicles. Looking closer at Figure 4f, tiny black spots were visible, matching the results of CLSM for DD. A possible explanation for these spots could be the agglomeration of curcumin after rupture and leakage of the fluidic liposomes during nebulisation. These results indicated the infeasibility of the fluidic formulation DD for nebulisation and were in accordance with the results of AFM, CLSM, DLS and measurements of the encapsulation efficiency.

### 3.5. Aerodynamic Properties

Aerosols are the products generated from drug solutions or dispersions during the process of nebulisation and their aerodynamic properties determine the location and intensity of the effect [57]. Measurements of the FPF according to monograph 2.9.18 of the 10th Edition of the European Pharmacopoeia were used to specify the aerodynamic characteristics after nebulisation with PARI VELOX^®^.

Suitable droplet size distribution, velocity and trajectory of an aerosol optimises the desired pulmonary deposition. Hence, it is important to know which parameters have an impact on these properties and which have none. Higher conductivity of the precursor fluid leads to a more efficient liquid breakdown [65]. Therefore, 20 mM HEPES pH 7.4 seems appropriate for the rehydration of lipid films as it facilitates a good output rate during nebulisation. However, the precise differences between the HEPES buffered liposomal formulations and ultrapure water regarding the output rate are shown in Section 3.1. Surface tension and reservoir volume do not have a relevant impact on aerodynamic properties, as described in the literature [65]. In addition, the vibrating mesh is moving too fast to give surface tension a chance to improve the liquid breakdown [65]. Since nanocarriers are mostly used to encapsulate small amounts of highly potent active ingredients, they are produced in small volumes. To ensure a successful therapy, it has to be certain that these small volumes get nebulised in a reliable manner, which again speaks for the suitability of vibrating-mesh nebulisers in this context, as the hydrostatic pressure on the nebuliser mesh does not affect the aerosol diameter [65]. Previous studies revealed that aerosol droplet sizes have to be between 1 and 6 µm for an optimal effect. Droplets larger than 6 µm may deposit in the mouth or throat, droplets smaller than 1 µm can be exhaled, both cases lead to ineffectiveness [67,68]. According to literature, with PARI VELOX^®^ up to 75% of the produced aerosol droplets are smaller than 6 µm and the average volumetric-median-diameter of the aerosol droplets is 3.6 to 4.4 µm [42]. The FPF measured in this study was defined by monograph 2.9.18 of the European Pharmacopoeia as droplets smaller than 6.4 µm and was stated as percentage ± standard deviation of active ingredient from the total nebulised amount of the latter. The liposomal formulation DC showed the best result with an FPF of 62.7 ± 1.6%, DT showed 59.5 ± 2.4% and DD 58.2 ± 1.4%. For the previously lyophilised liposomes, the FPF was 57.3 ± 3.8% for DT, 55.9 ± 6.2% for DC and 58.0 ± 5.4% for DD.

Overall, the FPF of all three nebulised formulations was relatively high and in the same range. This indicates that the composition and stability of liposomes does not affect the liquid breakdown and that 20 mM HEPES pH 7.4 provides a sufficient conductivity for nebulisation. Among the previously lyophilised liposomes, the stabilisation of DT seemed to be a slight advantage.

### 3.6. Mucous Membrane Compatibility

**Figure 5:**
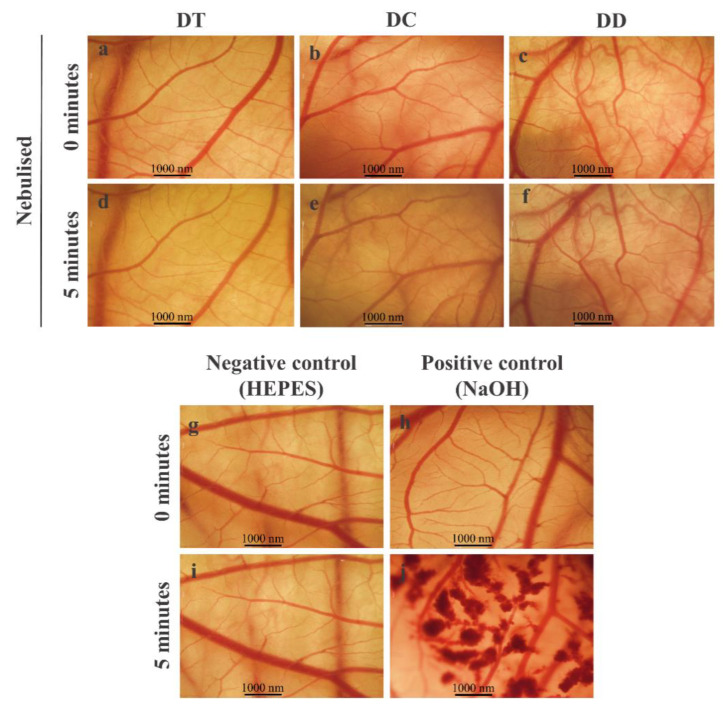
HET-CAM assay for mucous membrane compatibility of nebulised curcumin-loaded liposomes (**a**–**f**). HEPES 20 mM pH 7.4 served as negative control (**g**,**i**); 0.1 N NaOH as positive control (**h**,**j**)**.** Scale bars represent 1000 nm.

The mucous membrane compatibility of the nebulised curcumin-loaded liposomes was tested on the CAM and resulted in an irritation index of 0 for all three formulations. This means that none of the liposomal formulations caused a negative reaction, consequently mucosal intolerance. Therefore, the images were taken at the beginning as well as at the endpoint of the test (5 min) and show no changes (Figure 5). The positive control shows the occurrence of bleeding (Figure 5j).

### 3.7. In Vitro Cytotoxicity, Cellular Uptake and Migration

**Figure 6:**
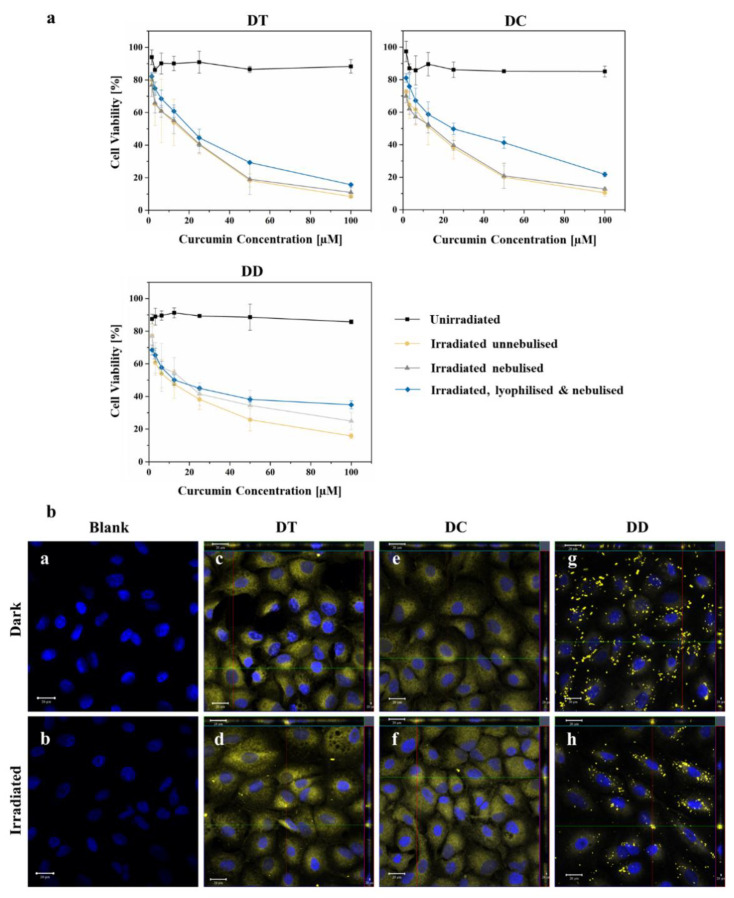
(**a**) MTT in vitro cytotoxicity assay of DT, DC and DD containing a curcumin concentration up to 100 µM. The figure shows a comparison of dark control, irradiated (IR) unnebulised, IR nebulised (PARI VELOX^®^) and IR lyophilised & nebulised curcumin-loaded liposomes. Irradiation always took place at 457 nm with a fluence of 6.61 J cm^−2^; (**b**) CLSM micrographs of A549 cells with nebulised curcumin-loaded liposomes containing a curcumin concentration of 100 µM. Dark and irradiated (457 nm, 6.61 J cm^−2^) samples were compared for the formulations: DT, (**c**,**d**); DC, (**e**,**f**); and DD, (**g**,**h**). Untreated cells were used for the blank micrographs (**a**,**b**). The cell nuclei were counterstained with 0.1 µg/mL DAPI and fixed with a 4% formaldehyde solution for all micrographs. Scale bars represent 20 µM.

To determine the photodynamic effect of the nebulised curcumin-liposomes, MTT cytotoxicity studies were made. They revealed low dark toxicities of 88.32 ± 4.14% cell viability for DT, 85.03 ± 3.30% for DC and 85.73 ± 1.26% for DD, which was within the acceptable range for all formulations [14,69]. The cell viabilities after treatment with irradiated nebulised liposomes were 10.99 ± 3.04% for DT, 12.76 ± 0.58% for DC and 24.90 ± 2.70% for DD (Figure 6a). All formulations showed a significant difference (*p* < 0.05) between dark control and irradiated nebulised samples, as well as between dark control and irradiated un-nebulised samples, respectively. This indicates an excellent cytotoxic efficacy and an improved nebulisation suitability of liposomal formulations containing tetraether lipids due to the strong adhesion forces within their lipid bilayer [62]. Besides, the ability of TEL to positively affect the intermembrane exchange of the active ingredient, thus enhancing the delivery [64], was also confirmed by these results. The difference between dark control and irradiated lyophilised and nebulised curcumin-liposomes was also significant (*p* < 0.05) for DT and DC, but not for DD. A decreasing cytotoxic effect from DT to DD was visible. Again, these results validate the improved stability of DT and DC liposomes towards nebulisation and the general eligibility of nebulised liposomes for pulmonary PDT. In addition, the results confirm the fact that curcumin is a suitable photosensitiser for a PDT of tumours [70,71].

Qualitative evidence of the internalisation of nebulised curcumin-loaded liposomes into A549 cells was given via visualisation by confocal laser scanning microscopy. After incubation with nebulised formulations containing 100 µM curcumin, a considerable accumulation of the photosensitiser was detected within the cells, which is evident from the z-stack images. Furthermore, for DT and DC a homogenous distribution of curcumin inside the cells was clearly visible (Figure 6b–f). Curcumin from the formulation DD appeared as crystal-like agglomerates (Figure 6b,g,h), probably due to its instability towards nebulisation resulting in a leakage of curcumin. AFM micrographs of DD (Figure 3e,f), recorded after nebulisation of the curcumin-loaded liposomes, showed small liposomal fragments which confirms the thesis of fluidic liposomes being unstable towards nebulisation. Results of the encapsulation efficiency confirm this thesis as well, as the value dropped from 85.1% to 57.5% (Table 3) for DD. Curcumin, originally encapsulated in the lipid bilayer of DD liposomes, was liberated and hence, given the opportunity to aggregate. Similar behaviour of ultra-deformable liposomes regarding fragmentation, decreasing encapsulation efficiency and aggregation of active ingredient occurred in the study of Elhissi et al. [40]. Although no difference in the cell appearance was observable in these micrographs, the assumption that the irradiated nebulised curcumin-loaded liposomes were the source of the cytotoxic effect (Figure 6a), seems very likely and is consistent with the literature [26].

One of the aims of encapsulating a drug in nanocarriers such as liposomes is to transport it to the site of action, in this study adenocarcinoma cells of the lung. For this purpose, the active ingredient must be taken up into the cells as a final step, which can take place by various mechanisms. The most important mechanism is endocytosis, which can play a significant role especially for hydrophobic drugs [72]. This active process can occur without external stimuli or can be induced by ligands [73]. Initially, an insertion is formed in the cell membrane for this purpose, in which extracellular substances or particles are trapped and which is subsequently internalized by constriction. Different endocytosis pathways can be distinguished; of relevance in this study were clathrin-mediated endocytosis on the one hand, and calveolae-mediated endocytosis on the other. Macropinocytosis as well as non-clathrin-non-calveolae-mediated endocytosis can also occur, but were not considered in this work.

**Figure 7:**
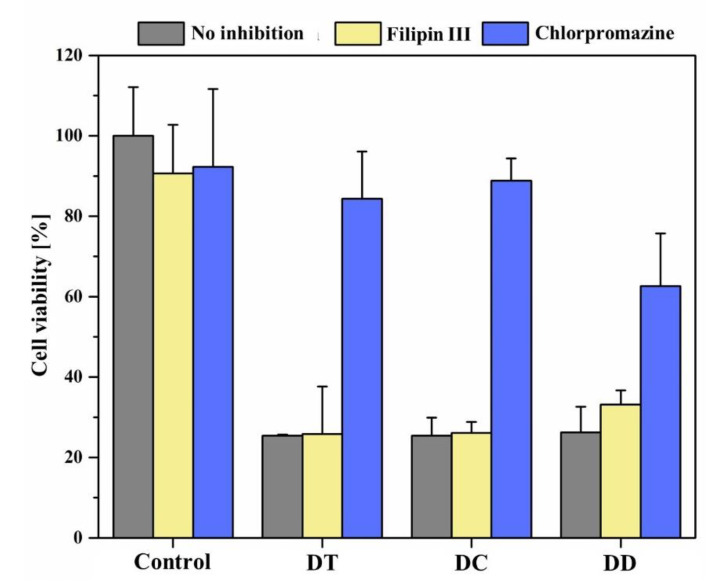
Determination of the endocytotic uptake mechanism of nebulised curcumin liposomes after irradiation with a fluence of 6.61 J cm^−2^ at λ = 457 nm. The endocytosis pathways were either inhibited by filipin III or chlorpromazine or were not inhibited. The incubation time of liposomes was 4 h, and untreated cells represented the 100% value of cell viability.

To identify the preferred endocytosis pathway of the nebulised curcumin liposomes, appropriate controls were first used to ensure that the inhibitors alone did not reduce cell viability. It can be seen that they have little effect on cell viability (Figure 7). Chlorpromazine served as an inhibitor of clathrin-mediated endocytosis, and filipin III inhibited caveolae-mediated endocytosis. When liposomes were applied after preincubation with filipin III, DT and DC after irradiation showed that the effects without inhibition were comparable to the effects after inhibition of caveolae-mediated endocytosis. This implies that liposome uptake into cells was possible in both cases and suggests that this endocytosis pathway is not the preferred one. The crossmatch after preincubation with chlorpromazine confirms this, as cell viability is significantly increased. Blockade of clathrin-mediated endocytosis inhibited liposome uptake to a large extent. These results agree with previous studies, according to which liposomes with a diameter of up to 200 nm are mainly taken up via the formation of so-called “clathrin-coated pits” and subsequent endocytosis [74]. In contrast, the DD formulation also appears to be taken up via caveolae-mediated to a lesser extent, with clathrin-mediated endocytosis still being preferred (Figure 7). Again, this could be related to the altered size distribution of these fluid liposomes.

In addition, the influence of PDT with nebulised liposomes on the migration behaviour of A549 cells was determined in this study. This was done by means of an “in vitro scratch assay”, a method based on the observation of an artificially created scratch in the confluent cell monolayer. The cells at the edge of this gap move towards the open area, which happens with the aim of closing the gap as quickly as possible until new cell-cell contacts have been created [75].

**Figure 8:**
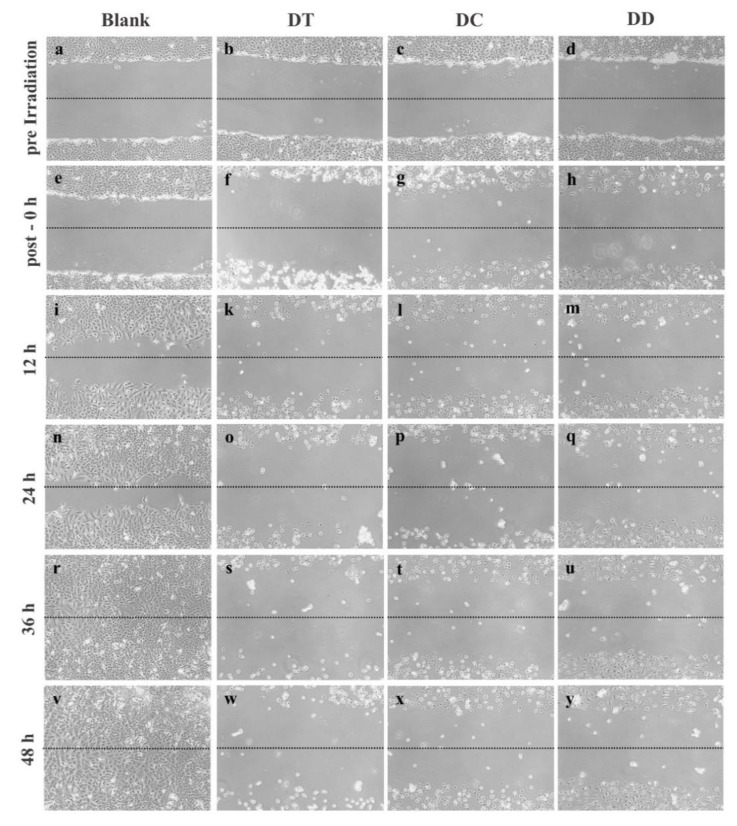
Microscopic images of the A549 cells during 48 h of the “in vitro scratch assay”. The images a, e, i, n, r and v show the natural migration of untreated cells (blank) compared to cell migration after PDT treatment with nebulised curcumin liposomes (b,e,d,f,g,h,k,l,m,o,p,q,s,t,u,w,x,y).

The results of this assay show that the natural migration of untreated cells closed the gap in the cell monolayer within 48 h (Figure 8a,e,i,n,r,v). In comparison, it can be seen that the gap in the cells treated with PDT does not close within this time period. The effects on the cell migration behaviour seems to be more distinct for DT and DC, in agreement with the previous results. From the time of irradiation onwards, especially in the case of DT, many cells can be seen to have detached from the bottom of the well (Figure 8f). This can be recognised by the round shape and a bright glow of the cells and is usually a sign of their death. After treatment with the formulation DD, there is still a slight migration or growth of the cells (Figure 8h,m,q,u,y). An explanation for the good migration inhibition of all three formulations could be the fact that curcumin can, in addition to the ROS generation during PDT, inhibit matrix metalloproteinases 2 and 9 in A549 cells, which play a known role in migration, invasion and angiogenesis of these cells [76].

## 4. Conclusions

Summing up, curcumin was taken up via clathrin-coated pits and visualised inside the cells. The good efficacy of nebulised curcumin liposomes with membrane stabilisation on cell viability and migration confirms their eligibility to improve pulmonary PDT.

## Figures and Tables

**Table 1:** Lipid compositions of the liposomal formulations. The lipids 1,2-dipalmitoyl-sn-glycero-3-phosphocholine (DPPC), tetraether lipids (TEL), 1,2-dioleoyl-sn-glycero-3-phosphoethanolamine (DOPE) and cholesterol (Chol) were used.

Abbreviations	Formulation	Molar Ratio (%)
DT	DPPC:TEL	90:10
DC DD	DPPC:Chol DPPC:DOPE	70:30 75:25

**Table 2:** DLS analysis of DT (DPPC:TEL), DC (DPPC:Chol) and DD (DPPC:DOPE). Curcumin-loaded liposomes nebulised via vibrating-mesh (PARI VELOX^®^) and air-jet nebulisation (PARI BOY^®^ SX) were compared with each other, respectively. Unnebulised curcumin-loaded liposomes were used as control. Values of particle size represent the distribution by intensity. Independent formulations were used to measure the triplicates and all results were expressed as mean ± standard deviation.

	Lipid Compositions	Size (nm) ± SD	PdI ^1^ ± SD	ζ-Potential (mV) ± SD
	DT	129.7 ± 3.2	0.19 ± 0.08	−13.53 ± 2.42
CUR-liposomes	DC DD	109.4 ± 2.4 94.4 ± 5.9	0.14 ± 0.09 0.26 ± 0.11	−5.61 ± 0.79 −2.97 ± 0.81
Nebulised (PARI VELOX^®^) CUR-liposomes	DT DC DD	131.1 ± 3.0 116.4 ± 3.2 99.1 ± 5.2	0.21 ± 0.06 0.18 ± 0.07 0.30 ± 0.09	−16.33 ± 1.95 −3.74 ± 0.64 −2.86 ± 0.59
Nebulised (PARI BOY^®^ SX) CUR-liposomes	DT DC DD	129.5 ± 2.8 113.3 ± 3.5 100.7 ± 4.7	0.20 ± 0.08 0.21 ± 0.09 0.32 ± 0.08	−15.54 ± 2.13 −5.13 ± 0.92 −3.11 ± 0.76
Lyophilised & nebulised (VELOX^®^) CUR-liposomes	DT DC DD	149.6 ± 4.1 127.7 ± 4.8 132.1 ± 5.1	0.46 ± 0.10 0.47 ± 0.13 0.57 ± 0.13	−37.89 ± 4.43 −11.47 ± 2.38 −9.62 ± 2.09

^1^ Polydispersity index.

**Table 3:** Encapsulation efficiency (EE) and loading capacity (LC) of DT (DPPC:TEL), DC (DPPC:Chol) and DD (DPPC:DOPE) liposomes. The results in this table were calculated according to Equations (1) and (2) in Section 2.8 for liposomes containing a curcumin concentration of 0.1 mg per 1 mg lipids. Unnebulised curcumin-loaded liposomes were used as control.

	Lipid Compositions	EE (%) ± SD	LC (%) ± SD
	DT	93.9 ± 8.2	1.9 ± 0.1
CUR-liposomes	DC DD	88.4 ± 12.4 85.1 ± 15.9	1.7 ± 0.1 1.7 ± 0.1
Nebulised (PARI VELOX^®^) CUR-liposomes	DT DC DD	80.0 ± 12.8 62.5 ± 12.3 57.5 ± 16.6	1.6 ± 0.1 1.3 ± 0.1 1.2 ± 0.1
Lyophilised and nebulised (VELOX^®^) CUR-liposomes	DT DC DD	74.6 ± 10.9 60.3 ± 9.5 31.2 ± 11.7	1.5 ± 0.1 1.2 ± 0.1 0.6 ± 0.1

## Data Availability

The data presented in this study are available on request from the corresponding author.
